# Conceptualization and Measurement of Flow in a Chinese Blended English as a Foreign Language Learning Context

**DOI:** 10.3389/fpsyg.2022.839267

**Published:** 2022-01-27

**Authors:** Xin Wang, Chenguang Huang

**Affiliations:** ^1^School of Humanities and Foreign Languages, Xi’an University of Science and Technology, Xi’an, China; ^2^School of English Studies, Xi’an International Studies University, Xi’an, China

**Keywords:** blended EFL learning, foreign language flow, factor analysis, positive psychology, FLFS psychometrics

## Abstract

This study takes a holistic view of flow and anti-flow experiences as interactive subsystems in blended English as a foreign language (EFL) learning and examines the dynamic complex construct in the field of instructed second language acquisition (ISLA). We first rephrased the 22-item *Classroom Flow Questionnaire* (CFQ) to better reflect the context of blended EFL learning. The modified CFQ was then administered to 661 first language Chinese EFL learners. A final 14-item *Foreign Language Flow Scale* (FLFS) was developed based on results from a series of reliability (e.g., item analysis, internal consistency, and test-retest reliability) and validity (e.g., construct validity, convergent validity, discriminant validity, and criterion validity) tests. Both exploratory and confirmatory factor analysis results have demonstrated that foreign language learning flow is a three-dimensional construct involving *Enjoyment*, *Boredom*, and *Anxiety*, thus conceptualizing and validating flow as a continuum with both positive and negative ends. Moreover, participants reported that they experienced the lowest degree of enjoyment, while with respect to the negative flow, they almost experienced similar degree of boredom and anxiety. The present study contributes to the development of the conceptual framework for flow in ISLA as well as constructive pedagogical implications for L2 researchers and educators. Suggestions for future research are also provided.

## Introduction

Based on the theoretical foundations of Flow Theory and Positive Psychology (PosPsy) in the field of instructed second language acquisition (ISLA), the interaction of flow and anti-flow as multifaceted subsystems in FL learning has attracted scholarly attention in the traditional setting of English as a foreign language (EFL; Linely et al., 2006; Schüler, 2012; Czimmermann and Piniel, 2016; MacIntyre et al., 2016; Dewaele and MacIntyre, 2019; Dewaele and MacIntyre, 2020). It has been noted, however, that the issue facing blended learning environments is relatively unexplored in the literature.

As a complex, multifaceted, and demonstrative construct (Shin, 2006), a blended learning model combines traditional teaching methods with online or web-based learning (Osguthorpe and Graham, 2003; Neumeier, 2005; So and Brush, 2008; Bowyer, 2017; Albiladi and Alshareef, 2019) in an effort to create a more “accessible, flexible, active, interactive, encouraging, and inspiring” teaching and learning environment (Zhang and Zhu, 2018, p. 268) where motivated learners are more likely to fully participate in and beyond class (Marsh, 2012; Albiladi and Alshareef, 2019). In fact, blending learning environments are inherently associated with positive and negative emotions (D’Mello, 2013) because benefits and difficulties coexist in that environment, resulting in learners’ recognition or underestimation of its effects (Albiladi and Alshareef, 2019).

Though scholarly inquiry has addressed quantitative data reflecting EFL learners’ flow state, there is no reliable and valid construct that is applicable to different learning environments and contexts. The assessment of EFL learners’ flow experience has not been sure in ISLA (Li et al., 2021), especially in blended learning environments. It is thus essential to assess EFL students’ flow characteristics within a blended learning setting to help SLA researchers identify learners’ levels of flow and develop a holistic view of learners’ emotions in EFL classrooms.

## Literature Review

### Flow in the EFL Context

Flow, one of the founding fields of positive psychology (Gilman et al., 2009; Czimmermann and Piniel, 2016; MacIntyre et al., 2016), is generally recognized as an optimal emotional state where people are fully engaged with whatever they are doing at the time, and their deep involvement leads to better performance (Csíkszentmihályi, 1975, 1990; Csíkszentmihályi and Rathunde, 1993) both at work (Csíkszentmihályi and LeFevre, 1989) and in school (Schüler, 2007). As researchers and teachers misinterpreted “optimal” as “desirable outcomes,” the nature of flow as a harmonious balance between positive and negative emotions (Dewaele and MacIntyre, 2019) was overlooked. Thus, the destructive end of the construct has faded into the shadow of positivity across a wide range of disciplines including SLA, which undermines Csíkszentmihályi’s (1990) argument that the bright side of flow is accompanied by a dark side.

The bright side of flow, or flow-enhancing experience, entails a balance between task challenge and learner skill, where tasks are addressed with skills appropriate to the situation (Dewaele and MacIntyre, 2019). In contrast, flow barriers, or negative flow experiences, are always associated with emotions, such as anxiety, boredom (Csíkszentmihályi, 1990), apathy, and worry (Nakamura and Csikszentmihalyi, 2002), where lies the most unfavorable blend of low challenges and low skills, that is, apathy (Dewaele and MacIntyre, 2019). Relative to apathy, a state of boredom arises when skills increase but challenges do not, while worry and anxiety occur when challenges increase but skills do not. Indeed, the positive flow channel lies between anxiety and boredom, as medium-high skills encounter increasing challenges and vice versa (Nakamura and Csikszentmihalyi, 2002). Though Csíkszentmihályi and LeFevre (1989) stated that “in such context, (people) report feeling more active, alert, concentrated, happy, satisfied, and creative” (p. 816), they did not overestimate the positive outcomes, as multifaceted flow experiences may not only provoke positive results, but also negative ones (Schüler, 2012).

However, most research centers on the positive aspects of the concept, particularly in ISLA (Egbert, 2003; Aubrey, 2017a,b, 2021; Li et al., 2021; Liu and Song, 2021), with fewer explicitly exploring both flow and anti-flow in EFL learning (Czimmermann and Piniel, 2016; Dewaele and MacIntyre, 2019). It has only been in recent years that the concept of flow has been empirically examined, using a multimethod approach that integrated quantitative and qualitative data to explore the intricate relationships between flow-enhancing and flow-prohibiting experiences, as well as flow’s complex interactions within itself.

### Flow Assessment Measures in the EFL Context

In terms of assessing flow in EFL contexts, the seminal study was conducted by Egbert (2003) who recruited 13 students from a high school in Spain and assessed them on seven tasks under fieldwork conditions. To identify features of flow-enhancing tasks, the researcher used a mixed-method approach including observations, interviews, and perceptions surveys. The researcher used a questionnaire developed by Webster et al. (1993) to assess learners’ perceptions of their experience and reported desirable internal consistency (Cronbach’s *α* = 0.82; Plonsky and Derrick, 2016). Based on self-reports, observations, and post-task interviews, the researcher found that learners experienced positive flow in the FL classroom where task design was a facilitator. Furthermore, the researcher categorized task-specific flow into four basic dimensions: (1) the challenge-skills balance resulting in task success and enhanced learner motivation, (2) focused attention featuring consciously attending to language input, (3) learner interest cultivated through completing clear, interesting, and achievable tasks, and (4) learner control or autonomy in FL learning. Indeed, those factors were extracted from the flow theory of Csíkszentmihályi (1975) and Csíkszentmihályi and LeFevre (1989). In addition, an adapted 14-item FPQ with four dimensions can be considered preliminary in ISLA. Despite the fact that learners described quite similar experiences of positive flow in the study, researchers should be aware not to generalize his research findings across many contexts, given the unstable reliability and validity of the adapted scale.

As well, Czimmermann and Piniel (2016) conducted an extensive quantitative study on FLF, sampling 85 first-year English majors at a Hungarian university to explore the correlations between the underlying dimensions (i.e., *Classroom Challenge-Skills Balance* and *Classroom Interest*). Of note, the researchers asserted that negative and positive emotions may occur simultaneously in a state of flow, which further extended flow research. Further, in response to Egbert’s (2003) enumeration of four task-based flow components and Oláh’s (2005) scale of flow, the researchers adapted a CFQ and a *Task-specific Flow questionnaire* (TFQ) in order to collect data on learners’ classroom and task-specific flow experiences. The study showed that university EFL students scored higher than secondary school students in classroom flow, which was consistent with Oláh’s (2005) assertion that flow antecedents, such as learners’ focused attention, interest, and adjusted challenge-skills balance, are more likely to be acquired at the university level. At the end of the study, the researchers concluded that classroom and task-specific flow were significantly interrelated in both flow and anti-flow experiences. Despite the contributions made by CFQ and TFQ, we noticed that the CFQ did not reach an acceptable level of internal consistency (*α* = 0.70; 0.74 ≤ *α* < 0.82 should be deemed acceptable, as recommended by Plonsky and Derrick, 2016).

In addition, Aubrey (2017b) used a quasi-experimental design to reveal Japanese EFL learners’ inter-cultural and intra-cultural task-based flow experiences. The study involved 63 first-year Japanese undergraduates, 42 of whom were domestic students enrolled in intensive English program, and 21 of whom were international students seeking a bachelor’s degree or attending a short-term academic exchange program. The researcher used the FPQ directly from Egbert (2003) and analyzed data from that questionnaire recorded on a 7-point Likert scale (coded from “1” = absolutely untrue to “7” = absolutely true). In a meta-analysis of reliability, Cronbach’s coefficients indicated that the flow scale and two subscales of *Interest* (*α* ≥ 0.80) and *Challenge-Skills Balance* (*α* ≥ 0.88) were reliable, but *Control* (0.44 ≤ *α* ≤ 0.70) and *Attention* (0.60 ≤ *α* ≤ 0.73) were not. Accordingly, the quantitative analyses led the researcher to conclude that the inter-cultural context was more conducive to flow than the intra-cultural one, and there was a weak positive correlation between flow and L2 engagement in the form of interactivity for the inter-cultural group.

After that, Dewaele and MacIntyre (2019) sampled 232 multilingual students across all levels of education to investigate flow-related learner variables (linguistic and socio-biographical profiles), and flow and anti-flow levels by using the newly developed *Flow Measure* that included eight questions determining the amount of time participants experienced positive flow and negative flow, and concluded with an open-ended question asking about classroom experiences that participants had enjoyed. An convergent parallel study design was utilized in this study, which revealed that as: (1) the proportion of time in a state of positive flow quantitatively and qualitatively outweighed that of negative flow, (2) learner variables, such as numbers of languages studied, relative standing among peers, and years of FL study, were significantly and positively correlated with positive flow, but not negative flow, (3) learners’ level of education did not affect their states of flow, and (4) a significant positive correlation was found between learners’ age and the proportion of time in a positive flow, but a significant negative correlation in a negative flow. Despite the valuable results of the research, it deserves attention that the reliability tests of the *Flow Measure* demonstrated an acceptable design of items reflecting flow experiences (Cronbach’s *α* = 0.81), if not as inadequate as anti-flow items (Cronbach’s *α* = 0.60). Thus, the research findings need to be interpreted carefully.

In contrast to the literature reviewed above, Chinese researchers in ISLA (Li et al., 2021; Liu and Song, 2021) did contribute to empirical explorations into flow by identifying relevant variables in technology-based or online FL learning environments with a variety of digital games and online activities. Based on Shin’s (2006) conceptual model of online learner’s flow experience, Li et al. (2021) and Liu and Song (2021) developed two distinct versions of flow questionnaires that differed in item numbers and underlying dimensions. Further, Liu and Song (2021) differentiated their research from that of Li et al. (2021) mainly in the research design. Specifically, while the former triangulated their quantitative research findings with additional interviews on dubbing experience to identify differences in flow between high achievers and low achievers in the FL, the latter relied solely on quantitative analyses, including factor analysis and structural equation modeling, to address learners’ vocabulary problems. Also, both studies demonstrated an inadequate construct reliability. More specifically, Li et al. (2021) reported only borderline reliability coefficient for the subdimension Feedback (Cronbach’s *α* = 0.794), while Liu and Song (2021) did not report consistent high reliability (Cronbach’s *α* ranging from 0.484 to 0.918). Despite that, these findings support a complex dynamic relationship between flow antecedents, flow experiences, and flow consequences. In more detail, Li et al. (2021) used 291 valid questionnaires to establish their model and found that learner and contextual variables were likely to positively influence flow experiences, such as concentration, intrinsic motivation, and enjoyment, which in turn affected flow outcomes, such as perceived learning and satisfaction. Researchers constructed nine dimensions for their 30-item questionnaire, including *Challenge-Skills Balance*, *Clear Goal*, *Feedback*, *Playability*, *Concentration*, *Intrinsic Motivation*, *Enjoyment*, *Perceived Learning*, and *Satisfaction*. In a study involving 235 junior high schoolers, Liu and Song (2021) concluded that participants showed medium-to-high level of flow in all three categories of flow and that learners’ linguistic proficiency affected the flow antecedents, but not the outcomes. Together, these two empirical studies, situated in Chinese EFL contexts outside of face-to-face classroom settings, offered insight into ways to enhance learner achievement in China.

### Qualitative Research on Foreign Language Flow

In the landmark qualitative study on EFL learners’ state of flow, Aubrey (2017a) sampled 63 Japanese EFL learners in a medium-sized private university in Japan to determine the likelihood of their positive flow experiences and the pattern of change in the strength of flow dimensions over five oral tasks for inter-cultural and intra-cultural interactions. The researcher conducted a quasi-experimental study in two stages over 11 weeks. This study uncovered six dimensions of flow: *Challenge-Skill Balance*, *Control*, *Interest*, *Enjoyment*, *A Sense of Accomplishment*, and *Attention*, significantly extending Egbert’s (2003) four-dimension model. Additionally, the researcher used the content analysis approach to analyze learner diary entries, which provided a plausible framework for future qualitative research on emotions.

### Motivation of the Present Study

Flow is observed during the performance of language tasks both in Western (Egbert, 2003; Czimmermann and Piniel, 2016; Dewaele and MacIntyre, 2019) and Eastern contexts (Aubrey, 2017a,b; Li et al., 2021; Liu and Song, 2021). Despite inspiring the research reported here, these studies limit the generalization of research on flow from different environments, since different contexts (Aubrey, 2017a) and environments may introduce additional dimensions to flow. Thus, it is necessary to develop an instrument that incorporates items that target each dimension of flow in order to address the calls of Hwang and Fu (2018) for future studies to concentrate on learners’ affective experiences of learning in the new way of learning, and of Aubrey (2017b, p. 15) to “develop the instrument by adding items specific to the research context that target each dimension of flow and attempt to rigorously validate the questionnaire.” To that end, this study tried to answer the following three questions:

- How reliable and valid is the newly adapted version of the FLFS in Chinese undergraduates in relation to their EFL learning in a blended environment?
- How do the undergraduates conceptualize foreign language flow?
- What are the overall and dimensional profiles of the students’ foreign language flow?

## Materials and Methods

### Local Context

The participants in this study were enrolled in a non-English-major undergraduate course titled “College English.” There were two types of textbooks used, including one that targeted listening and speaking, and the other that addressed comprehensive English proficiency, including listening, speaking, reading, and writing in English, and most importantly, translating between Chinese and English. The course was taught primarily face-to-face in physical classrooms using technology-based apps, such as *Vocabulary Spam* or *Cidaren*,^1^ *Welearn*,^2^ and *Danmupai*^3^ to preview theme-related keywords, video clips, and text-based audio clips, and to stimulate classroom interaction and to review lectures.

### Participants

Given the relatively easy access to data, we employed a convenience sampling procedure in June 2021 to recruit a total of 661 participants from the same university in Northwest China. After an extensive review, six invalid questionnaires were eliminated from the pool of samples. Thus, the global sample included 655 (99.1%) non-English-major undergraduate students in Year 1. We created two samples to evaluate the psychometric properties of the scales. Sample 1 was comprised of 502 students who participated in the main study, which included several confirmatory factor analyses. They came from 16 different classes at the same university. They were 306 (61%) male participants and 196 (39%) female participants. The mean age was 19.36 (*SD* = 1.014). They have learned English for 10.52 (*SD* = 2.985) years on average. A total of 400 (79.7%) were from the discipline of Natural Science and 102 (20.3%) from Humanities and Social Sciences. Sample 2 consisted of 153 students who participated in exploratory factor analysis (EFA) and then retested with a 2-week interval. They were from three different classes at the same university. There were 103 (67.3%) male participants and 50 (32.7%) female participants. The mean age was 19.39 (*SD* = 0.988). Participants did not report the length of time they spent learning English. In addition, 433 out of the total sample participated in the criterion validity test.

The first author distributed the online questionnaire to students as an English teacher at the university that recruited the participants for this study. In order to reduce bias, the second author provided participants with information regarding the research goals, possible influences, and basic research procedures before administering a questionnaire. Specifically, the data collected would be used only for research purposes, and their English teachers would not have access to any personal information. All students provided informed written consent after the information session. To ensure that the items were understood correctly, we used a Chinese version of the questionnaire. After translating the FLFS from English to Chinese, a professor of psychology cross-checked the translated version and modified its wording. Finally, we posted the electronic questionnaire online and collected the data through *Wenjuanxing*.^4^

### Instrument

We collected quantitative data through a composite questionnaire. The questionnaire consisted of two sections, namely, a sociodemographic section inquiring about participants’ background (e.g., name, age, gender, major, and number of years in FL learning) and the FLFS items asking about flow and anti-flow experiences during FL learning.

### Foreign Language Flow Scale

The CFQ (Czimmermann and Piniel, 2016) was adapted from the *Flow Perceptions Questionnaire* (FPQ; Egbert, 2003). There was a mix of positive and negative wording in the items. In this study, all items were reworded to better fit the current blended EFL learning context, thus forming the FLFS. An example item is “I continuously feel that things are going smoothly [both on and off line].” Accordingly, the modified FLFS had two sub-dimensions: *classroom challenge-skills balance* and *classroom interest*, encompassing 11 items respectively. The 22 items were arranged on a 5-point Likert scale from “1” (not at all or never) to “5” (very much or almost always). We validated the 22-item scale in the target sample and further modified it in exploratory and confirmatory factor analyses. Also, no FLFS item was positively phrased so that it displayed both the bright and dark aspects of flow. Further, to ensure a better understanding of the items, we reordered the item “Most of the time, what I have to do is an exciting challenge for me” so that it fits better with the anchors.

### Flow Perceptions Questionnaire

In the present study, the FPQ (Egbert, 2003) was used as a criterion scale of the FLFS, following the practice of Czimmermann and Piniel (2016) who examined the correlations between task-specific flow and classroom flow, as well as task-based boredom, apathy, and state anxiety. The FPQ was originally designed by Webster et al. (1993) to measure learners’ perception of flow and thereafter modified by Egbert (2003) in EFL learning contexts. The modified FPQ consisted of 14 items arranged on a 7-point Likert scale ranging from “1 (Strongly disagree)” to “7 (Strongly agree).” Generally, it is recognized as having a four-factor structure and measuring four indicators of flow, namely, *Interest*, *Control*, *Focus*, and *Challenge*. In order to reduce the inaccuracies in the distinction between anchors, the present study used an adapted 5-Likert scale and demonstrated good reliability (*ɑ* = 0.785, *n* = 433).

Each item has been translated into Chinese for ease of understanding. The translation was completed by the second author with expertise in second language acquisition and checked by a professor in psychology. Discrepancies were discussed until everyone reached an agreement on the exact translation.

### English Final Examination Paper 2021

The examination consisted of four parts: Listening (20 points), Reading Comprehension (40 points), Cloze Test (10 points), Translation (15 points), and Writing (15 points). The examination was completed within 2 h and administered in June 2021 to approximately 4,000 undergraduate students in the same university. We used the examination paper to measure participants’ English achievement.

### Data Analysis

We conducted several tests to assess various psychometric properties of the FLFS at both global and dimensional levels. With the first step, we examined its item discriminating validity in SPSS (Statistical Package for the Social Sciences) 26.0. Following that, we conducted confirmatory factor analyses in Mplus 8.3 with Sample 1 (*n* = 502) using the estimator of maximum likelihood, robust standard errors, and mean adjusted chi-square test statistics (MLM) to refine and confirm the original two-factor structure of the FLFS when the chi-square value expanded due to multivariate abnormality of the collected data. We carefully examined the model fit using indices, such as Chi-Square, RMSEA, CFI/TLI, and SRMR, as recommended by Kline (2016). Table 2 presents their benchmarks.

As a next step, we performed EFA using Geomin oblique rotation and the MLR estimator with extracted factor limits to four in order to test the presumed unidimensionality of the new flow model and to refine it by reducing items. Afterward, another set of confirmatory factor analyses revealed a three-factor, 14-item FLFS. As well, we assessed the ability of the observed items to represent a specific latent factor and the degree to which they differ from each other in the measure as convergent and discriminant validity. This helped us confirm the goodness of fit of current data to the global FLFS. Specifically, the refined scale was subjected to tests of convergent validity, discriminant validity, and criterion validity (*n* = 433), followed by tests of internal consistency and test-retest reliability (*n* = 153). Further, we assessed internal consistency by calculating Cronbach’s alpha, and composite reliability, and rest-retest reliability. Both Cronbach’s alpha and composite reliability were applied to estimate construct reliability in this study since Cronbach’s alpha tended to severely underestimate internal consistency (Urbach and Ahlemann, 2010) by assuming that items were equally weighed, while composite reliability took into account that indicator items had different loadings. In the end, we performed a descriptive analysis, using the data retrieved from the previously validated and confirmed FLFS.

## Results

This section presents the results in response to the three research questions.

### Psychometric Properties of the FLFS

Our validation and reliability tests were conducted on a sample of Chinese EFL learners in a blended environment to confirm and refine the scale.

### Item Analysis

Item analysis was first conducted to determine item discrimination. We created the upper 27% (*n* = 120) and lower 27% (*n* = 232) groups based on their total scores in the 22 items. An independent-sample *t-test* was performed at item level. The results showed a significant difference on each item between the two groups (all at *p* < 0.01 level), indicating that all the items in the scale were appropriate for further analysis.

Afterward, item-total correlation analyses were conducted between the score of each item, those of the subscales, and the global score. Table 1 summarizes the results. As determined by the benchmark correlation coefficient (*r* = 0.30) between each item and the global scale (Field, 2016), Item 22 was eliminated (averaged *r* = 0.089).

**Table 1:** Correlation matrix of each item, subscales, and the global scale.

	Item	Interest	Challenge-skills balance	Global
Interest	Q1	0.690	0.268	0.540
	Q2	0.732	0.298	0.580
	Q4	0.675	0.326	0.563
	Q8	0.632	0.230	0.486
	Q9	0.252	0.567	0.450
	Q10	0.716	0.314	0.580
	Q11	0.271	0.326	0.331
	Q12	0.731	0.363	0.615
	Q16	0.256	0.426	0.377
	Q18	0.622	0.250	0.491
	Q21	0.437	0.148	0.330
Challenge-skills balance	Q3	0.213	0.580	0.435
	Q5	0.101	0.544	0.352
	Q6	0.423	0.376	0.445
	Q7	0.546	0.326	0.489
	Q13	0.624	0.400	0.574
	Q14	0.142	0.472	0.337
	Q15	0.239	0.630	0.477
	Q17	0.183	0.597	0.427
	Q19	0.204	0.618	0.451
	Q20	0.551	0.367	0.514
	Q22	0.010	0.164	0.094

### Construct Validity

Confirmatory factor analysis was then conducted to determine whether the original dual-factorial structure of classroom flow could also be supported by the data obtained in our current EFL sample. Following the benchmarks for assessing model fit (Kline, 2016), the first trial of CFA results indicated that the original two-factor model with 21 items was not supported (see Table 2 CFA1).

**Table 2:** CFA and EFA results of the 21-item and 14-item versions of foreign language flow scale.

Index	Benchmark	Research models			
		CFA1	EFA	CFA2	CFA3
No. of items		21-item	21-item	21-item	14-item
*χ* ^2^	Smaller is better	1842.81	251.139	505.310	144.933
df	Larger is better	209	150	186	74
Δ*χ*^2^	5>Δ*χ*^2^ < 1	8.82	1.67	2.72	2.42
CFI	>0.90	0.602	0.918	0.893	0.966
TLI	>0.90	0.558	0.886	0.879	0.958
RMSEA	<0.08	0.125	0.066	0.058	0.044
SRMR	<0.08	0.128	0.040	0.054	0.039

*χ*^2^ = Chi-square; Δ*χ*^2^ = Normed Chi-square = df/*χ*^2^.

**Figure 1:**
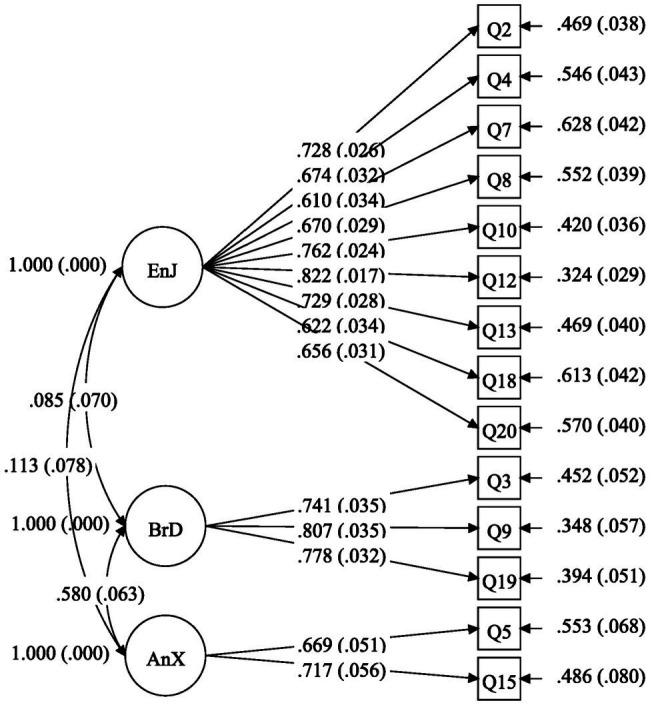
The three-factor model of foreign language flow.

As the first CFA result was not satisfactory, we conducted an EFA in Mplus 8.3 using Geomin oblique rotation and the MLR estimator in order to confirm a competitive model with good fit. The results supported a tri-factorial structure of the adapted CFQ (see Table 2 EFA). In order to refine and confirm the newly identified structure, we then carried out confirmatory factor analysis (CFA) on Sample 1 (*n* = 502). Results from the first trial of CFA indicated that the three-factor flow model was not supported by data from the current study (see Table 2 CFA2). Thereafter, seven items (1, 6, 11, 14, 16, 17, and 21) were removed owing to their low factor loadings (<0.60) or high modification indices (Item 1 = 56.654). In a subsequent CFA, a construct consisting of 14 items was further examined, and the model fit was found to be excellent (see Table 2 CFA3). This resulted in a three-factor model with both positive and negative flow constructs being adequately supported. The results of this study demonstrated that the FLFS can be appropriately modeled as a 14-item, three-factor construct (see Table 2 CFA3 and Figure 1).

### Convergent Validity

Aside from the overall construct validity of the FLFS, validity at the subscale level was also evaluated in terms of the convergent validity (Wu, 2010). The average variance extracted (AVE) was applied to determine the extent to which each item for a particular factor was convergent compared with items reflecting other factors. Table 3 presented acceptable (AVE > 0.36) to excellent (AVE > 0.5) model fit for each subscale (Fornell and Larcker, 1981).

**Table 3:** Item reliability, composite reliability, convergent validity, and discriminant validity of the FLFS and its subscales.

Sub(s)cale	Item	Parameters of significance test				Composite reliability	Convergent validity	Discriminant validity		
		Std.	S.E.	*Z*	*P*	CR	AVE	EnJ	BrD	AnX
FLFS	14 items					0.936	0.513			
Enjoyment	Q2	0.728	0.026	28.236	0.000	0.896	0.490	**0.700**		
	Q4	0.674	0.032	21.29	0.000					
	Q7	0.610	0.034	17.698	0.000					
	Q8	0.670	0.029	23.052	0.000					
	Q10	0.762	0.024	31.866	0.000					
	Q12	0.822	0.017	47.315	0.000					
	Q13	0.729	0.028	26.422	0.000					
	Q18	0.622	0.034	18.488	0.000					
	Q20	0.656	0.031	21.498	0.000					
Boredom	Q3	0.741	0.035	21.083	0.000	0.819	0.602	0.085	**0.776**	
	Q9	0.807	0.035	22.817	0.000					
	Q19	0.778	0.032	23.975	0.000					
Anxiety	Q5	0.669	0.051	13.125	0.000	0.649	0.481	0.113	0.580	**0.694**
	Q15	0.717	0.056	12.887	0.000					

*Bold numbers are the subscales’ square roots of AVEs. Other discriminant validity numbers are the correlation coefficients. EnJ, Enjoyment; BrD, Boredom; and AnX, Anxiety*.

### Discriminant Validity

In addition, we examined the discriminant validity of each subscale by comparing the square root of each subscale’s AVE with correlation coefficients between each subscale. According to Table 3 and Figure 1, the square root of AVE for each subscale was higher than its correlation coefficients with other subscales, indicating excellent discriminant validity for each subscale.

### Criterion Validity and Predictive Validity

Lastly, validity is confirmed by comparing the newly modified 14-item FLFS with relevant scales in the literature. In accordance with the approach taken by Czimmermann and Piniel (2016), the criterion validity was assessed in relation to the well-established FPQ (Egbert, 2003). As shown in Table 4, the scores of students’ responses in the FLFS and the FPQ were significantly correlated at both the global and dimensional levels, with small to large effect sizes (Plonsky and Oswald, 2014), providing evidence of a close correlation between foreign language classroom flow and task-related flow.

**Table 4:** Correlation matrix of the *Foreign Language Flow Scale* and other measures.

(Sub) Scale	FPQ (*n* = 433)	FL achievement (*n* = 502)
FLFS	0.576***	0.204***
Enjoyment	0.630***	0.230***
Boredom	−0.217***	0.001
Anxiety	−0.035	0.076

***p < 0.001.

Table 4 demonstrates that both global flow and its positive flow factor were significant predictors of participants’ performance on the English test, with small effect sizes.

### Reliability

We examined both the internal consistency and the test-retest reliability by using Cronbach’s alpha, composite reliability (CR) results, and correlation coefficients. According to Table 3 and Table 5, the CRs for the global FLFS, as well as its three subscales (*Enjoyment*, *Boredom*, and *Anxiety*), were 0.936, 0.896, 0.819, and 0.649 (see Table 3), while their alphas were 0.785, 0.895, 0.839, and 0.675, respectively (see Table 5), indicating desirable reliability.

**Table 5:** Internal consistency and 2-week test-retest reliability of scale and subscales.

(Sub) Scale	ɑ (*N* = 655)	*r* (*n* = 153)	*M_T_* (*n* = 153)	*SD_T_*	*M_R_* (*n* = 153)	*SD_R_*
FLFS	0.785	0.533***	45.51	4.47	45.34	3.86
Enjoyment	0.895	0.783***	33.11	4.73	32.88	4.76
Boredom	0.839	0.625***	6.92	2.26	7.11	2.11
Anxiety	0.675	0.653***	5.49	1.69	5.38	1.48

***p < 0.001; T stands for test; and R stands for retest.

Additionally, a total of 153 students participated in the second questionnaire survey using the new 14-item FLFS at an interval of 2 weeks (Wu, 2010). Their responses in the two surveys were significantly related to each other, with medium to high effect sizes (see Table 5).

### The Three-Factor Conceptualization of Foreign Language Flow

According to the factor analysis results from CFA, the initial two-factor structure of flow identified in Hungarian EFL context (Czimmermann and Piniel, 2016) was confirmed as the positive side of flow in the context of blended EFL education, and the subscales of negative flow were established by another round of EFA and CFA. Indeed, foreign language flow was characterized by a mixture of positive and negative experiences. More specifically, it was a three-dimensional construct consisted of *Enjoyment*, *Boredom*, and *Anxiety*. Correspondingly, the factors were assessed by three subscales consisting of nine, three, and two items, respectively. The newly modified three-factor model comprehensively portrayed Chinese EFL learners in a blended learning environment as not only having an interest in learning English, having fun when their mastery of language matched the task challenge, but also feeling bored or anxious when tasks were either too easy or too demanding, and sometimes not able to concentrate on the task at hand.

### General Profiles of Chinese Undergraduates’ Level of Foreign Language Flow

We calculated the mean scores of each subscale within the FLFS construct, as well as their average scores. Table 6 provided an overview of the status of Chinese undergraduates’ foreign language flow at both global and dimensional levels. Paired-sample tests showed that the dimensions were significantly different from each other with large effect sizes (all *p* < 0.001, *df* = 654, *Enjoyment* – *Boredom*: *t* = 91.102, Cohen’s *d* = 7.125, effect size *r* = 0.963; *Enjoyment* – *Anxiety*: *t* = 109.795, Cohen’s *d* = 8.587, effect size *r* = 0.974; *Boredom* – *Anxiety*: *t* = 27.378, Cohen’s *d* = 2.141, effect size *r* = 0.731; Plonsky and Oswald, 2014), indicating they were relatively independent ingredients of the FLF construct except for a medium positive correlation between *Boredom* and *Anxiety* (*r* = 0.467^***^). In addition, according to the mean scores in Table 6 and their average scores (*Enjoyment: Mean* = 3.47, *SD* = 0.618; *Boredom: Mean* = 5.00, *SD* = 0.907; and *Anxiety: Mean* = 5.00, *SD* = 0.84), participants experienced negative flow more frequently than the positive flow in a blended EFL learning environment. Generally, enjoyment was a main contributor to students’ positive state of flow, whereas anxiety was a leading cause of their negative state.

**Table 6:** Status of Chinese undergraduates’ foreign language flow in blended learning (*N* = 655).

Factor	Min.	Max.	Mean	*SD*	Skewness	*SE*	Kurtosis	*SE*
FLF	1.00	5.00	45.81	6.51	0.821	0.095	1.607	0.191
Enjoyment	1.00	5.00	31.24	5.56	0.235	0.095	0.243	0.191
Boredom	1.00	5.00	15	2.72	0.110	0.095	−0.317	0.191
Anxiety	1.00	5.00	10	1.68	−0.003	0.095	0.234	0.191

## Discussion

The current study provides, for the first time, an FL-specific measurement for student flow in a blended learning environment. A series of validity (construct validity, convergent validity, discriminant validity, and criterion validity) and reliability (item analysis, internal consistency, and test-retest reliability) test results confirm that the current 14-item FLFS adapted from the 22-item CFQ has desirable psychometric properties and could be applied in future research examining EFL learning in a blended environment.

To address our first two research questions, we assessed both the reliability and validity of the modified FLFS model in the context of blended EFL learning in China, as well as the robustness of its factor structure, and thereby developed a framework for understanding foreign language flow of Chinese EFL learners in blended learning. Indeed, there have been a few studies that examine the factor structure of the FLES with respect to both flow and anti-flow experiences (Czimmermann and Piniel, 2016; Dewaele and MacIntyre, 2019), as well as its replications, both cross-culturally and cross-contextually, in a Chinese EFL technology-based learning context (Li et al., 2021; Liu and Song, 2021). An indication of excellent model fit was observed (*χ*^2^ = 144.933, Δ*χ*^2^_(74)_ = 2.42, CFI = 0.966, TLI = 0.958, RMSEA = 0.044, SRMR = 0.039) in the present study when examining Chinese samples who were enrolled in a blended EFL class. Further, this study revealed a three-dimensional structure of the FLFS with *Enjoyment*, *Boredom*, and *Anxiety* dimensions, of which the Enjoyment dimension encompassed the widest range of sub-dimensions, such as *Interest*, *Focused Attention, Challenge-Skills Balance, Control,* and *Motivation*. This factor structure resembled that of TFQ with a small-scale sample of Hungarian EFL learners (Czimmermann and Piniel, 2016) and revealed a more nuanced distinction in the manner we integrated the positive flow sub-dimensions of *CFQ-Interest* and *CFQ-Challenge-Skills Balance* to an umbrella dimension *FLFS-Enjoyment*. In addition, we found similar findings to four other studies conducted in the Asian EFL context showing that (*Focused*) *Attention* and *Enjoyment* dimensions underlay the construct of foreign language flow in a Japanese EFL context (Aubrey, 2017a,b) and that *Concentration* and *Enjoyment* dimensions underpinned the construct in a technology-based Chinese EFL context (Li et al., 2021; Liu and Song, 2021). Despite this, our factor structure differs from the previous ones in that we have not only identified *FLFS-Boredom* and *FLFS-Anxiety*, but also integrated *Interest* (*Focused*)*, Attention, Challenge-Skills Balance, Control,* and *Motivation* into *FLFS-Enjoyment*, rather than separating them from *FLFS-Enjoyment*, as in Aubrey’s (2017a) and Li et al.’s (2021) studies. Generally, these distinctions can be interpreted as evidence that a variety of learning contexts or environments may contribute to a diversified pattern eliciting flow.

Following construct validity tests, we conducted a series of reliability tests to examine its internal reliability by calculating Cronbach’s alpha, composite reliability (CR), and test-retest reliability. We found Cronbach’s alpha coefficients of 0.785 for the global FLFS, 0.895 for *Enjoyment*, 0.839 for *Boredom*, and 0.675 for *Anxiety*, indicating a desirable internal consistency (*ɑ* > 0.70) except for the subscale *Anxiety* as suggested by Nunnally and Bernstein (1994). Further, the modified FLFS had higher internal consistency than the *Flow Measure* (*α* = 0.81 for flow and 0.60 for anti-flow) by Dewaele and MacIntyre (2019) and the original two-factor CFQ (*α* = 0.70) by Czimmermann and Piniel (2016). In addition, the FLFS was proven to be reliable by the CRs at both global (CR = 0.936 > 0.70) and dimensional levels as well as participants’ retest results on the FLFS.

Unsurprisingly, we found a positive predicative effect of positive foreign language flow, in the form of *Enjoyment*, on L2 achievement, with a small effect size (*r* = 0.230, *p* < 0.001), and a negative correlation between task-based foreign language learning flow and FL classroom boredom (*r* = −0.217, *p* < 0.001). On one hand, foreign language enjoyment has been extensively examined both in traditional (Dewaele and MacIntyre, 2014, 2016; Jin and Jun Zhang, 2018; Li et al., 2018) and in online classroom settings (Wang et al., 2021), revealing its strong positive correlation with FL achievement in ISLA (Jin and Jun Zhang, 2018; Li et al., 2019). The present study corroborates their robust relationship in a blended learning context by establishing enjoyment both as a core component of flow and a predictor for achievement. On the other hand, foreign language boredom has kindled academic attention in ISLA (Pawlak et al., 2020a, 2021; Li, 2021; Li and Dewaele, 2021) and several studies have documented its potential negative impact on FL learning and achievement (Kruk, 2019; Pawlak et al., 2020b; Li, 2021). Contrary to enjoyment and boredom, flow does not receive as much attention in relation to FL learning despite its theoretical relevance to optimal human mental functioning, other psychological characteristics (such as focused attention, engagement, anxiety, boredom, apathy, and relaxation) and achievement (Egbert, 2003; Li et al., 2021; Liu and Song, 2021). This suggests the necessity for studying students’ foreign language classroom flow, and the connections it has with various learning aspects and second language achievement. The need for such investigations is particularly apparent in Chinese EFL context, where FL education varies considerably in its delivery and interaction (Wang et al., 2021) and is nowadays oriented toward blended learning approaches.

Our third research question examined Chinese EFL learners’ level of flow and anti-flow in blended learning in terms of its discrete dimensions. To our best knowledge, the new FLFS is the only measure specially designed to assess this construct in a Chinese-blended EFL learning context. As discussed in the Results, our participants reported much boredom (*Mean* = 5.00, *SD* = 0.907) and anxiety (*Mean* = 5.00, *SD* = 0.84) than enjoyment (*Mean* = 3.47, *SD* = 0.618). Interestingly, our Chinese undergraduates are involved in not only positive learning experiences of intensively focusing on activities, gaining interest in their novel course delivery and interaction, perceiving control over the process and possible outcome, and ultimately acquiring personal confidence and motivation to learn English, as well as negative experiences, such as feeling bored with under-challenging tasks, being upset by too demanding tasks that challenged their language proficiency and becoming easily distracted from blended learning activities. Therefore, we have sufficient evidence to estimate the new construct as a valid measure with three distinctive but slightly overarching dimensions that capture an overall picture of Chinese EFL learners as experiencing both positive and negative flow during their blended learning. Moreover, we observe that despite the advantages blended learning provides for drawing learners’ attention and facilitating classroom interactions (Liu, 2013), students also lose control over and are sometimes incapable of focusing on FL tasks (Riel et al., 2016). Thus, we propose that blended learning is a facilitator rather than an absolute determinator for successful FL learning. Teachers and practitioners should prioritize a balance between task challenge and students’ language proficiency to mitigate the negative effects of boredom and anxiety on FL learning, and promote students’ control over FL tasks both online and offline to further strengthen their confidence and motivation in a positive learning atmosphere.

## Limitations, Directions for Further Research, and Conclusion

Although this study extended previous research into three dimensions of flow and further analyzed their correlations under the conceptual framework of concurrent flow-enhancing and flow-prohibiting experiences, there are several methodological limitations in our research design. First of all, we have already mentioned that the sample size of this study was small, which means we cannot argue that our participants represent a typical sample of Chinese EFL learners. Second, this retrospective approach may fail to capture learners’ fluctuating consciousness and autotelic experiences, although it is generally regarded as a more useful method to ask participants about a previous experience than to have them complete the scale immediately after engaging in an activity that may not result in a feeling of flow (Jackson and Marsh, 1996).

We agree with Egbert (2003, p. 508) that “there is no objective way to measure flow precisely,” indicating that “a variety of instruments should be used to satisfactorily capture such a slippery concept” (Aubrey, 2017b, p. 8). However, we do not claim that the FLFS instrument is the only or even the best instrument to examine flow. Indeed, it is open to question whether these findings are applicable cross-culturally to other populations or cross-contextually to traditional face-to-face classroom settings. Further, although the three dimensions of the FLFS were analyzed separately for ease of interpretation, we realized that a number of sub-factors spanned the three dimensions and interacted with each other. Thus, future researchers should take a holistic approach to address the latent variables of a construct.

Despite the fact that flow is a complex concept in the literature concerning intrinsic motivation, learning autonomy, and various emotions, few studies have examined flow in the field of ISLA, let alone combining it with anti-flow, regardless of whether the second or foreign language learning occurs in a traditional face-to-face classroom, in a web-based classroom, or in a blended learning environment. Therefore, it is necessary to conduct more empirical research on the combination of flow and anti-flow in a single study in order to expand the scope of flow theory and its applications in the instructed learning.

## Data Availability Statement

The raw data supporting the conclusions of this article will be made available by the authors, without undue reservation.

## Author Contributions

XW designed the research, processed the data, and wrote the whole manuscript. CH collected the data and revised the manuscript. All authors contributed to the article and approved the submitted version.

## Funding

The authors disclosed receipt of the following financial support for the research, authorship, and/or publication of this article. This study was funded by the Teaching Reform Project (2021) awarded to the first author by Xi’an University of Science and Technology (# JG21106).

## Conflict of Interest

The authors declare that the research was conducted in the absence of any commercial or financial relationships that could be construed as a potential conflict of interest.

## Publisher’s Note

All claims expressed in this article are solely those of the authors and do not necessarily represent those of their affiliated organizations, or those of the publisher, the editors and the reviewers. Any product that may be evaluated in this article, or claim that may be made by its manufacturer, is not guaranteed or endorsed by the publisher.

## Footnotes

^1^*Vocabulary Spam* or *Word Spam* is a mobile platform for learning English words. It provides its users with a variety of services, for example, presenting students with new words along with their pronunciation alphabets, colloquial phrases, and context-based sentences, and allowing teachers to launch word quizzes or assess how well a student has accomplished a task.

^2^*Welearn* is a web-based English learning platform that can be accessed *via* mobile devices and a formal website allowing students to receive instant feedback on their performance, and teachers to monitor students’ progress. Shanghai Foreign Language Education Press developed and owns this resource.

^3^*Danmupai* is a standalone version of computer-assisted classroom interaction software which aims at promoting student participation. Upon registration, the teacher will display a QR code on the screen, and the students will scan it using their mobile devices. Then, students will be able to respond to questions by typing out their responses and send them through the app. Their responses will appear on the screen for their fellow students and teachers to comment on.

^4^*Wenjuanxing* provides online questionnaire services including questionnaire design, distribution, and data analysis. It provides users with a platform to include an electronic questionnaire, and to distribute the questionnaire by sharing a link, or QR codes for participants to complete on their smart devices and computers.

## Appendix

### The Adapted 14-Item English Version of the Foreign Language Flow Scale (FLFS)

Please think about the blended learning class for this semester and share your experiences in the following way: Please tick (√) before an item to indicate how much you agree with the statement.[Not at all/ Not really/ Neutral/ Somewhat/ Very much]

1. I am immersed in what I do in and outside class.
2. I get bored with some learning tasks.
3. I enjoy the experience of make full use of what I can to complete a task.
4. I get nervous with tasks in face-to-face class and online learning platforms.
5. I constantly feel that things are going smoothly during blended learning.
6. I am willing to do the tasks even if it was not required.
7. The tasks are uninteresting for me.
8. I’m generally engaged in the tasks we do.
9. I am intensely interested in those tasks.
10. The tasks are exciting and challenging for me, and I enjoy being able to complete them.
11. When the demand for a task is too high, I feel nervous.
12. I know exactly what I am supposed to do.
13. The tasks in blended learning pattern are dull for me.
14. I consider that I can meet the requirements for tasks during blended learning.
